# Nanoscale Self-Assembly for Therapeutic Delivery

**DOI:** 10.3389/fbioe.2020.00127

**Published:** 2020-02-25

**Authors:** Santosh Yadav, Ashwani Kumar Sharma, Pradeep Kumar

**Affiliations:** Nucleic Acids Research Laboratory, CSIR Institute of Genomics and Integrative Biology, Delhi, India

**Keywords:** self-assembly, nanostructures, amphiphilicity, polymers, small molecules, drug delivery

## Abstract

Self-assembly is the process of association of individual units of a material into highly arranged/ordered structures/patterns. It imparts unique properties to both inorganic and organic structures, so generated, via non-covalent interactions. Currently, self-assembled nanomaterials are finding a wide variety of applications in the area of nanotechnology, imaging techniques, biosensors, biomedical sciences, etc., due to its simplicity, spontaneity, scalability, versatility, and inexpensiveness. Self-assembly of amphiphiles into nanostructures (micelles, vesicles, and hydrogels) happens due to various physical interactions. Recent advancements in the area of drug delivery have opened up newer avenues to develop novel drug delivery systems (DDSs) and self-assembled nanostructures have shown their tremendous potential to be used as facile and efficient materials for this purpose. The main objective of the projected review is to provide readers a concise and straightforward knowledge of basic concepts of supramolecular self-assembly process and how these highly functionalized and efficient nanomaterials can be useful in biomedical applications. Approaches for the self-assembly have been discussed for the fabrication of nanostructures. Advantages and limitations of these systems along with the parameters that are to be taken into consideration while designing a therapeutic delivery vehicle have also been outlined. In this review, various macro- and small-molecule-based systems have been elaborated. Besides, a section on DNA nanostructures as intelligent materials for future applications is also included.

## Introduction

**FIGURE 1:**
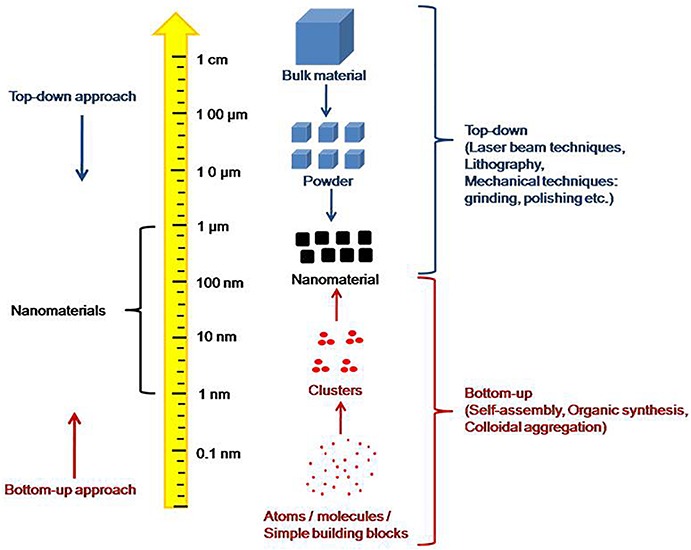
“Top-down” and “bottom-up” approaches of self-assembly.

Supramolecular self-assembly has recently attracted the attention of the researchers worldwide to generate nanostructures and nanomaterials bearing unique physical and chemical properties. The organization of molecules in these nanoassemblies has made it possible to design and develop new devices that can interact with the living cells and generate the response. These are not only being focused as important components in the emergence of cellular life, but also as materials that can be used in huge applications ranging from diagnostics and sensing to biomaterials, bioelectronics, energy generation, catalysis, drug delivery, and nanocomposites (Busseron et al., 2013; Du et al., 2015; Habibi et al., 2016; Xing and Zhao, 2016). Mainly, two strategies, viz., top-down and bottom-up, are being followed for the fabrication of nanostructures (Figure 1). The earlier one involves the carving out of the final nanostructure with a defined shape and size from a larger block of matter. As a result, the strategy does not require atomic level control. Alternatively, the later approach involves building up of the desired nanostructures from the basic components by the processes of molecular recognition and self-assembly, which is basically derived from the interactions of basic units to form well-organized structures. Therefore, the atomic or molecular level control is possible in the later approach over the formation of nanostructures by manipulating the structures of self-assembling molecular units.

## Self-Assembly

Self-assembly is the spontaneous molecular arrangement of the disordered entities of molecules into ordered structures resulting from specific local interactions among the components themselves (Mendes et al., 2013; Mattia and Otto, 2015; Stoffelen and Huskens, 2016). Formation of the most of the biological nanostructures is the outcome of the self-assembly such as construction of cell membranes by assembly of phospholipid bilayers, helical structure of DNA, folding of polypeptide chains, etc. The interaction of a ligand with its receptor is also attributed to self-assembly (Haburcak et al., 2016; Azevedo and da Silva, 2018). It also accounts for the development of molecular crystals, self-assembled monolayers, phase separated polymers, and colloids (Busseron et al., 2013; Mendes et al., 2013; Du et al., 2015; Mattia and Otto, 2015; Habibi et al., 2016; Haburcak et al., 2016; Stoffelen and Huskens, 2016; Sun et al., 2017; Azevedo and da Silva, 2018). In fact, molecular self-assembly is a natural process which is very essential in the emergence and maintenance of life. Synthetic molecules like amino acids, oligo- and polypeptides, polymers, dendrimers, and π-conjugated compounds have been considered as the primary focus used for building up nanostructures, such as nanotubes, nanofibers, micelles, and vesicles (Buerkle and Rowan, 2012; Correa et al., 2012). Moreover, self-assembly of small molecules as building units is a useful strategy for the formation of structure-controlled materials (Ariga et al., 2019). Likewise, DNA-based nanomaterials have shown their potential in diagnostics and therapeutic delivery.

The process of self-assembly plays a key role in the design, synthesis, and development of newer nanomaterials (Whitesides and Boncheva, 2002).

• Self-assembly is centrally important to living materials, e.g. a cell consists of a wide variety of complex structures, viz., lipid biomembranes, protein aggregates, folded proteins, structured nucleic acids, molecular machines, etc., which have shown the propensity of self-assembly.
• It helps in acquiring regular structures of materials, viz., molecular crystals, liquid crystals, and semicrystalline and phase-separated polymers.
• It also happens in large molecules, which has opened up newer avenues for their use in material sciences and delivery applications.
• It offers the most simple and versatile strategy for developing nanostructures.

Thus, self-assembly has exhibited a profound impact in a wide range of fields, viz., physical, chemical, and biological sciences, materials and biomedical sciences, and manufacturing. Besides, the concept has provided opportunities to develop new materials and components of life through the exchange of ideas and methodologies among these fields.

## Classification of Self-Assembly

**FIGURE 2:**
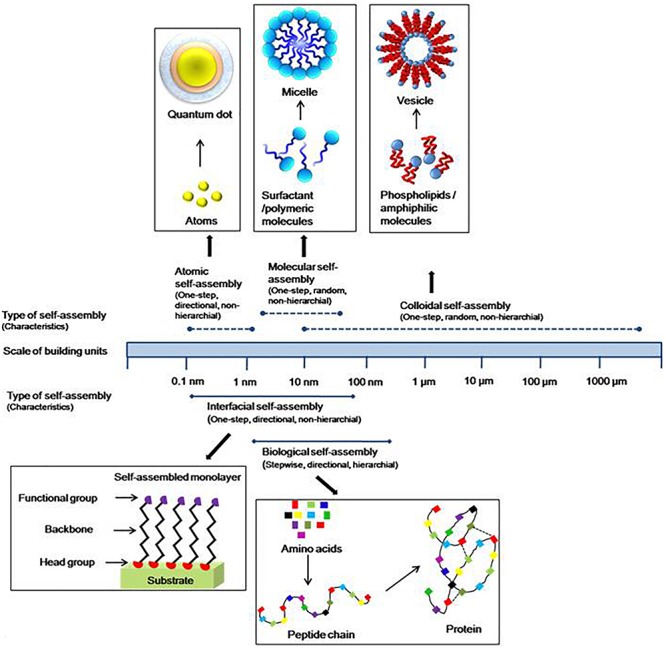
Atomic, molecular, colloidal self-assemblies based on size or nature of building units, and biological, interfacial on the basis of system where the self-assembly occurs. The length range is of structural units.

The term self-assembly was initially used by the researchers in different fields and subsequently, it was adopted by the chemists to describe the ordered arrangement of the molecules. Now, it has applied to materials of any size (from small molecules to galaxies) in the world around us (Xing and Zhao, 2016). Recently, the strategy has been shifted to synthesis of molecules which can be manipulated at the molecular level. This has become possible due to integration of chemistry, biology, and material science. Based on the size and nature of building blocks, self-assembly can be classified into three main categories, i.e. atomic, molecular, and colloidal self-assemblies (Figure 2). A variety of building blocks have been embraced in the term “self-assembly.” The process of self-assembly not only covers bulk materials, but also it can apply to two-dimensional systems, i.e. surfaces and interfaces. Thus, on the basis of the systems and where it occurs, it can be classified as biological or interfacial. Further, based on its processing, it can be categorized as thermodynamic or kinetic self-assembly. Atomic, molecular, biological, and interfacial self-assemblies are covered under thermodynamic processes, while colloidal and some interfacial self-assemblies come under kinetic ones. Among these types of assemblies, atomic and biological self-assemblies are directional while others are random or non-directional such as colloidal, molecular, and interfacial self-assemblies. Self-assembly involving large building units can also be responsive to one or the other external stimuli, viz., gravity, magnetic field, flow, electric field, surrounding media, etc.

Thus, as a result of self-assembly, spontaneous association can lead to generation of ordered structures in a range from angstrom to centimeter of different sizes and shapes. Historically, the concepts of self-assembly have come from the investigation of molecular/biological processes.

## Types of Interactions in Self-Assembly

**FIGURE 3:**
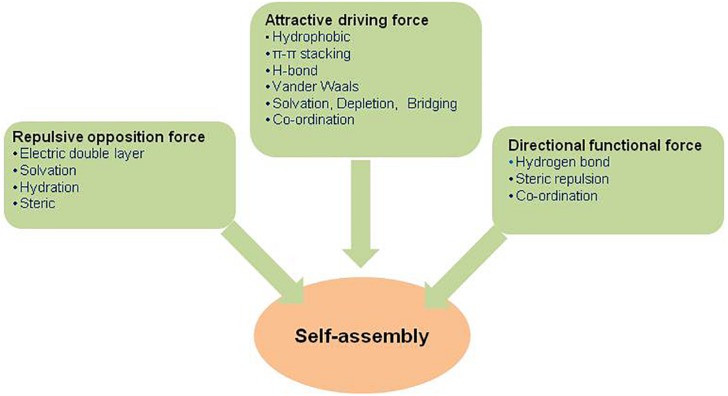
Three classes of distinctive forces involved in self-assembly.

Basically, the types of interactions that involved in the self-assembly processes occurring at colloidal, molecular, or atomic length scale are usually fragile and long range in contrast to chemical forces (Mendes et al., 2013). These are mainly non-covalently linked via van der Waals forces, hydrophobic, electrostatic, hydrogen bonding, π-π aromatic stacking, metal coordination, etc., which are normally weak (2–250 kJ/mol) individually in comparison to covalent linkages (100–400 kJ/mol) but together, if present in adequate numbers, they form very stable self-assembled structures and the shape, size, and functionality of the final assembly are administered by their fine balance (Mendes et al., 2013). Self-assembly between molecular units occurs when they interact with one another through a balance of usually weak and non-covalent intermolecular forces (Lee, 2008; Genix and Oberdisse, 2018). These interactions play a significant role in the alignment of molecular units in an ordered structure. These interactions are the main force that facilitates self-assembly of the units. Besides, the directionality and functionality of self-organized structures are determined by other functional interactions or forces (Figure 3). All these non-covalent interactions stabilize the self-assembled structures under different environmental conditions. Moreover, exhibition of completely new type of behavior as well as unique physical and chemical properties by self-assembled nanostructures have made them of special interest to researchers and scientists worldwide (Xing and Zhao, 2016). The distinctive intermolecular forces important in molecular self-assembly are given below (Mendes et al., 2013).

### van der Waals Interactions

van der Waals interactions consist of attractive or repulsive forces between molecules which operate at moderate distances. These forces arise from dipole or induced dipole interactions at the atomic and molecular level (Lee, 2008). These are strong in vacuum or if there is no medium between two molecules. If a medium (such as water) comes between the two molecules, these forces are reduced because of dielectric screening from the medium. Obviously, this screening effect is particularly strong for water due to its high dielectric constant. The energy of the van der Waals interactions is around 1 kJmol^–1^ whereas a covalent bond has a binding energy of around 150 kJmol^–1^ or more (hydrogen bonds, for comparison, have typical energies of around 50 kJmol^–1^). Overall, at atomic and molecular levels, van der Waals interactions are predominantly attractive, while, under certain conditions, these can also be repulsive (particularly, at short range).

### Electrostatic Interactions and Electric Double Layer

Electrostatic interactions occur between two charged atoms, ions, or molecules, which can be either - attractive or repulsive forces, depending upon the sign of charges. These interactions are quite strong and act even at long range (upto ∼ 50 nm), however, decrease gradually with distance. Ionic self-assembly is straightforward and considered to be a reliable method for the organization of polyelectrolytes, charged surfactants, peptides, and lipids (Lee, 2008; Mendes et al., 2013). These forces originate from electrostatic interactions and impart a strong effect on many self-assembly processes. Further, these forces act as balancing interactions along with hydrophobic interactions, which result in the finite size and shape of self-aggregated structures. Sometimes these interactions get added onto during self-assembly process. Self-assembly processes at the atomic scale involve the electrostatic interactions in air as well as in vacuum, while in solution, molecular and colloidal/mesoscale self-assembly processes occur.

The interfacial double layers are generally quite evident in systems having large surface area to volume ratio, such as porous or colloidal bodies with pores or particles, respectively, in the range of micrometers to nanometers. Layer by layer or double layer self-assembly plays an important part in several routinely employed materials, e.g. existence of homogenized milk, which is owing to coverage of fat droplets with a double layer that inhibits their agglomeration into butter.

### Hydrophobic Interactions

Hydrophobic interactions play a big role in understanding the process of self-assembly. These interactions occur in water due to poor dispersibility of the hydrophobic moieties. Interaction of a hydrophobic moiety with water can be elucidated using thermodynamic effects which result in the change in free energy, entropy, enthalpy, and heat capacity. These changes can be studied by the thermodynamic principle, Δ*G* = Δ*H* - *T*Δ*S*. When a hydrophobic substance interacts with water, the structure of water around that substance varies with the size and shape of the substance. This networking around the hydrophobic substance is called iceberg cluster or iceberg formation (Lee, 2008). The iceberg formation itself is not an entropic or enthalpic effect rather it depends upon the temperature and the geometry of the hydrophobic substance (Lee, 2008). Hydrophobic substances have been shown to exhibit extraordinary stronger interactions in aqueous phase as compared to the interactions in the gaseous state primarily because of van der Waals interactions. Therefore, due to poor dispersibility of hydrophobic moieties in water, they tend to form aggregates which ultimately result in self-assembly to generate micelles and lipid bilayers.

### Hydrogen Bonding

Hydrogen bonding constitutes the most attractive type of bonding in controlling inter- or intramolecular orientations in self-assembly. It also helps in understanding the variety of events in biological systems (Lee, 2008; Mendes et al., 2013). The strength of H-bonding varies from 10–50 kJmol^–1^, which indicates that this bonding has capability to provide sufficient stability to the self-assembled clusters. Basically, H-bonding occurs due to dipole-dipole attraction which takes place between a H-atom attached to an electronegative atom and an electronegative atom with lone pair of electrons present in the vicinity. Generally, it happens between H and O, F, and N. Strength of H-bonding is also affected by the surrounding medium, i.e. solvent. An additional feature of H-bonding is that it imparts stability as well as directionality to self-assembly. This property facilitates self-assembled structures to gain various morphologies useful for various biomedical applications.

### Aromatic π-π Stacking

Aromatic π-π stacking refers to another type of non-covalent interactions which are quite attractive to researchers for cooperative binding during self-assembly. It occurs between aromatic residues as they contain pi bonds. These interactions have been found to be of considerable importance in DNA and RNA molecules (nucleic base stacking), folding of polypeptide/protein chains, template-directed synthesis, materials sciences, and molecular recognition (Bissantz et al., 2010). Large polarizabilities and a significant quadrupole moment, generated by a particular shape and electronic properties of the aromatic ring systems, result in a set of preferred interaction geometries. As demonstrated by various theoretical and practical investigations, it has been well established that aromatic ring systems have tendency to form ordered clusters of four different types, viz., parallel displaced, T-shaped, parallel staggered, or Herringbone (Gazit, 2002). These geometries might be possibly potential minimum configurations in the Lennard–Jones–Coulomb empirical potential calculations. For interactions between two π systems, the predominant arrangements are the T-shaped edge-to-face and the parallel-displaced stacking arrangement. In proteins, the parallel-displaced stacking arrangement is observed more frequently. Stacking is potentially more favored between electron deficient aromatic rings rather than electron rich rings. Moreover, the alignment of positive and negative partial charges and molecular dipoles significantly affects the preference among the orientation of heteroaromatic rings. This becomes even more attractive when edge-to-face interactions are increased as a result of increased acidity of the interacting hydrogen atom. The effect is visible when a strongly electron withdrawing substituent in ortho or/and para position is introduced (Wheeler and Houk, 2009).

The steric constrains observed during the formation of the organized stacking structures have an essential role in the process of self-assembly that leads to the formation of supramolecular structures. Such π-π stacking interactions are responsible for stabilization of the tertiary structure of proteins, host–guest interactions, double-helix structure of DNA involved in core packing, and porphyrin aggregation in solution.

Gazit (2002) has also reported that π-π stacking interactions play a significant role in self-assembly of amyloid fibril formations. π-π stacking provides two important elements for the formation of these structures, (i) an energetic contribution that drives the self-assembly process thermodynamically and (ii) specific directionality and orientation that are driven by the set of stacking pattern (Gazit, 2002). This becomes more important because amyloid fibrils are well-defined supramolecular structures and a pre-determined pattern of stacking leads to formation of an organized structure. On analyzing a group of proteins with known structures having π-π stacking in them, it was noticed that a parallel displaced π-π stacking is the major organization of π-π interactions in proteins.

## Fabrication of Self-Assembled Aggregates

Self-assembly is a process that involves balancing between attractive driving force, repulsive opposition force, and directional force (Lee, 2008; Mendes et al., 2013; Genix and Oberdisse, 2018). Particularly, a sweet balance between attractive and repulsive forces initiates the formation of self-assembled aggregates, which is a random process and also shows non-hierarchical structures (Figure 3). Most of the colloidal and micellar systems fit in non-hierarchical type of self-assembly. Addition of directional force to the other forces, the self-assembly processes become directional. Moreover, the self-assembled aggregates in such cases usually show hierarchical structures that include biological and bio-mimetic systems.

### Micelles

In case of micelle formation by surfactant molecules, the attractive and repulsive forces guide surfactant molecules to come close enough to acquire an ordered structure (Lee, 2008; Xing and Zhao, 2016). The driving force that allows the formation of micellar system is the hydrophobic attraction while ionic repulsion and/or solvation force acts as the opposition force. As a result of this arrangement, at a certain position, the attractive and repulsive forces balance each other, which results in the formation of micelles. Concentration of the surfactant is the concentration that is required to form the first micelle (CMC). Addition of more amounts of surfactant molecules in bulk solution will result in the formation of additional micelles following the same force balance scheme. During this process, the size of the micelles remains invariable.

### Vesicles

Vesicles are sphere-shaped lamellar structures having a hollow aqueous core (Xing and Zhao, 2016). The formation of vesicles can be viewed as two-step self-assembly process in which amphiphiles first form a bilayer which then closes to form a vesicle. A number of amphiphilic organic compounds, varying from natural to synthetic, exhibit vesicle formation (Lombardo et al., 2015; Xing and Zhao, 2016). Natural phospholipids, amphiphilic polymers, and polypeptides capable of forming vesicles are called liposomes, polymerosomes, and peptosomes, respectively (Xing and Zhao, 2016). Among these classes of compounds, most of them are formed of a hydrophilic head and a lipophilic tail that induces formation of vesicles. During exposure to aqueous media, the hydrophilic head interacts with water while the hydrophobic tail contracts inside to minimize exposure to water. In this process, the lipophilic part of the amphiphile buries inside the bilayer and the hydrophilic part forms the interior and exterior positions exposed to aqueous environments. Differences in the arrangement of molecules lead to unilamellar or multilamellar vesicles with diameters in range from 20 nm to several micrometers according to the number of bilayers present in the newer structures formed.

In a self-assembly of amphiphiles into a vesicle or other types of structures, the volume ratio of the hydrophilic and lipophilic parts plays a significant role and it is a dominant factor which is now being applied for designing and development of vesicular structures.

In the equation, P = v/la,

“v” and “l” symbolize the volume and length of the lipophilic part, while “a” symbolizes the volume of the hydrophilic head. “P” values can help in speculating the morphology of the nanostructures and explaining the phase transitions.

If

P < 1/3, spherical micelles
1/3 < P < 1/2, worm-like micelles
1/2 < P < 1, vesicles
P = 1, planar bilayers
*P* > 1, inverted structures.

This theory was initially applied to surfactant systems but now it is being applied in studying self-assemblies of other kinds of amphiphiles that include amphiphilic block copolymers which follow the same principles as the small-molecule-based systems (Chu et al., 2013). Having both the hydrophilic interior and hydrophobic membrane, vesicles can be used to entrap both the hydrophobic as well as hydrophilic drugs at the same time. Liposomal vesicles have been well demonstrated to carry a wide range of therapeutic molecules and some of them are currently being used in clinical applications. Some recent papers focus on manipulating the size, shape, physical properties, and biodistribution of vesicles for drug delivery applications and emphasize the need of further control of these parameters of vesicles for therapeutic delivery applications (Zhao et al., 2017).

### Fibrillar Networks or Hydrogels

Hydrogels are 3-D continuous interpenetrated network of phases, the solid and the liquid phase (Lee, 2008; Mendes et al., 2013; Shang et al., 2019). The liquid phase of a hydrogel comprises of water while the solid phase is network structure in which nanofibers are formed via molecular self-assembly (i.e. molecular gelators). Fibers can be formed from self-assembled proteins, peptides, lipids, and hybrid amphiphiles. However, their formation is significantly dependent on hydrophobic–hydrophilic balance as it is essential for self-assembly. These nanofibers act as the matrices of a hydrogel. It also prevents the undesirable precipitation or dissolution of the hydrogelators (Du et al., 2015). The hydrophilic part of the molecule locates itself as the exterior portion of the nanofibers, which gets involved in hydrogen bonding with the surrounded water molecules making it certain that hydrophilic biomolecules such as drug molecules (small peptides) can be translated into hydrogelators. Such supramolecular structures interact with the target molecules/sites more efficiently than the native biomolecules thereby increasing their bioactivity. As a hydrogel contains ∼97% of water, still it behaves like a solid and can flow only when a shear force is applied. Generally, hydrogels display response to an external stimulus and undergo a phase transition upon its application because these are formed via the self-assembly of small molecules through hydrogen bonding and hydrophobic interactions which are quite weak interactions. Apart from this, the supramolecular hydrogels offer an added advantage that these are biocompatible and biodegradable, as well as resemble to extracellular matrices which help in design and synthesis of novel supramolecular hydrogelators as materials for biomedical applications. Hydrogel materials have been intended to synthesize for encapsulation and delivery of water soluble therapeutic molecules. There are many reports in literature which demonstrate the encapsulation and release of small hydrophilic molecules, proteins, and cells from the hydrogels (Narayanaswamy and Torchilin, 2019). Drug molecules can be entrapped into the networked structure during initiation of self-assembly process. Hydrogels, formed mainly by the process of self-assembly, are joined together through non-covalent crosslinking (covalent or physical hydrogels), which also determines its actual mechanical strength. The classification of the hydrogels can be made on the basis of their source (natural or synthetic), nature (degradable or non-degradable), networking (covalent or physical), and the nature of network (homopolymeric, copolymeric, interpenetrating networks, and double networks).

## Applications of Self-Assembled Materials

The term nanostructure generally refers to those materials/structures which have structured components with at least one dimension less than 100 nm. The properties (both physical and chemical) of nanostructures are markedly dissimilar from their monomeric unit or the bulk material having identical chemical composition. The main reason for this unique behavior at nano-scale is due to the appearance of new quantum effects as well as enhanced surface area to volume ratio (Dahman, 2017). As the nanostructures have higher surface area to volume ratio as compared to their conventional forms, they exhibit greater chemical reactivity and strength. These emergent properties exhibited at nano-scale have the potential for greater impacts in biomedical applications. Suitable modulation of the properties and response of nanostructures may result in the creation of new desired gadgets and technologies.

The area of nanobiotechnology for therapeutic delivery is flooded with new challenges as the demand for new medical therapies is increasing exponentially. Earlier, the nanomedicines were developed by reformulating the available drugs in nanostructures. With the development of nanomedicines, which have shown the potential to treat the diseases in a much better way, the demand for personalized medicines has grown up that requires the customization of the fabrication of the nanostructures with in-built desired properties (Cui et al., 2010). For this customization, an improved control over structure, composition, as well as function of the matter at molecular level is needed. To achieve this control at molecular level, self-assembly comes into picture which can play a very crucial role by adjusting various parameters such as size, shape, and surface chemistry mimicking the 3-D structure of biomacromolecules. Thus, novel nanomaterials can be produced with greater ease and economically by employing the tools of molecular self-assembly. Furthermore, diverse nanostructures with varied functionality can be produced by this process (Genix and Oberdisse, 2018). The great advantage of this scheme for the formation of nanosized structures is the structural control over the final self-assembled nanostructures which can be achieved by varying the monomer, its composition, and chemistry, by inducing environmental changes (solvents, temperature, pH, and co-assembling molecules), and changing the rate of self-assembly process (Dallin et al., 2019). The ultimate goal of these self-assembled nanostructures is to attain their required functions; whether these structures are thermodynamically stable or not. As discussed above, self-assembly easily provides the flexibility to develop newer materials with customized morphologies and preferred functionalities and thus provides better control over bulk properties of the resulting nanostructures. Hence, it is quite simple to presume the behavior of final assembly by controlling the structural changes in the constituent molecules. In recent past, a plethora of nanostructures have been produced using different biopolymers (proteins, carbohydrates, nucleic acids, etc.) which have further refined the concepts and knowledge of this process as well as enhanced the use of these self-assembled materials in diverse medical applications such as in fabrication of molecular devices, delivery systems, or scaffolds (Panda and Chauhan, 2014; Azevedo and da Silva, 2018; Lombardo et al., 2019). These systems have shown their promising potential, however, need more attention to address some limitations in terms of their *in vivo* stability which has hindered their safe use in human beings.

Self-assembled nanomaterials are being used for a very broad range of applications from fundamental to applied research, with striking implementations in biomedical sciences, information technology, and environmental sciences. Here, in this article, self-assembled nanostructures useful for biomedical applications have been the main focus, specially, drug delivery and gene delivery, so the subsequent part deals with these aspects (Busseron et al., 2013; Xing and Zhao, 2016).

### Drug Delivery

Therapeutic delivery is a very significant area to address concerns related to healthcare and medicine. Certain problems associated with the use of free drugs can be minimized by using the appropriate carriers for drugs such as stability issue of free drugs in biological system, short half-life, insolubility in aqueous environment, abnormality in biodistribution, and pharmacokinetics of the delivered drugs (Mohanraj and Chen, 2006). Controlled drug delivery has shown enhanced bioavailability of the therapeutic by avoiding their untimely degradation and improving their uptake, maintaining the therapeutic dose of the drug by controlling the kinetics of drug release, and reducing toxicity by targeting to desired sites/tissues. In this regard, nanoparticles have proved to be potential DDS due to their advantageous characteristics. Many positive aspects of nanoparticle-mediated delivery of therapeutics have been realized (Wang et al., 2018).

Nanoparticles as therapeutic delivery systems offer several advantages:

i. Particle size and surface properties of nanoparticles are amenable to manipulation to achieve drug targeting.
ii. Nanoparticles possess large surface to mass ratio; hence, they can bind, absorb, and carry large amounts of drug molecules.
iii. Nanoparticles can easily control the drug release during the process of uptake and internalization as well as at the intended site which helps in reducing side effects/toxicity of the drug.
iv. The rate of release of the drug as well as the degradation of a carrier can be manipulated by selecting appropriate matrix constituents. Moreover, the drug entrapment is quite high in nanoparticles and that too without any chemical reaction, rather these are retained via physical interactions which help in preserving the drug activity.
v. By attaching specific ligands onto the surface of the nanoparticles, site-specific targeting can be achieved.
vi. Nanoparticles can be delivered via different pathways such as parenteral, intra-ocular, intravenous, oral, nasal, etc.

### Criteria for the Designing of New Delivery Vehicles

Criteria for the designing of a new delivery vehicle are highly dependent on the therapeutics to be delivered and intended applications. Some of the common points, that are kept in mind while designing these vectors, are given below (Yu et al., 2016; Knauer et al., 2019).

i. The delivery vehicles need to be non-toxic, biocompatible, and biodegradable, and get readily eliminated from the body.
ii. These must possess high therapeutic loading efficiency, which would reduce the number of cycles of drug administration.
iii. These should not damage or modify the therapeutic agent during entrapment process.
iv. These vectors should be able to deliver the drug in a controlled fashion to allow consistently defined release profiles.
v. When administered, the carriers should be capable of providing stability to the therapeutics from degradation and neutralization by antibodies.
vi. These should be amenable to modification so that ligands could be attached for site-specific delivery. In this case, accumulation of the carriers at the desired site of action would facilitate the release of the therapeutic at the desired rate.
vii. These should be easily administered with little discomfort.
viii. The preparation of delivery system should be easy, reasonably simple, reproductive and cost-effective, and should be amenable to scale-up.

#### Polymers

A wide range of self-assembled polymeric nanostructures have been used for drug delivery, but biodegradability is essential to overcome side effects and toxicity to healthy tissues (Sofi et al., 2018; George et al., 2019). The self-assembled nanostructures are formed from both natural and synthetic polymers. Numerous self-assembled DDSs have been developed which have successfully encapsulated drug molecules to improve bioavailability, bioactivity, and controlled delivery, with some achieving clinical testing (Felice et al., 2014) and some of them have been launched for commercial purposes (Felice et al., 2014) (Table 1).

**TABLE 1:** List of nanoengineered polymers for drug delivery applications (Felice et al., 2014; Bobo et al., 2016; Patra et al., 2018).

**Product name**	**Carrier material**	**Drug/type of drug (Disease)**	**Approval year/phase**
Livatag^TM^	Poly(isohexyl-cyanoacrylate)	Doxorubicin/anthracycline (hepatocellular carcinoma)	Phase II
Lupron Depot^TM^	PLA	Leuprolid/peptidic (Prostate and breast cancer)	1989
Estrasorb^TM^	Lecithin	Estradiol/esteroide (Hot flushes during menopause)	2003
Risperdal consta^TM^	PLGA	Risperidone/dopamine antagonist (bipolar disorder Schizophrenia)	2003
Abraxane^TM^	Albumin	Paclitaxel/anthracycline (Breast cancer)	2005
Genexol-PM^TM^	PEG–PLA	Paclitaxel/anthracycline (Breast cancer)	Phase II
Adagen^TM^	PEG	Adenosine deaminase/peptidic (Severe combined immunodeficiency)	1990
Oncaspar^TM^	PEG	Asparaginase/peptidic (Leukemia)	1994
PEG-intron^TM^	PEG	Interferon α2b/proteic (Chronic hepatitis C)	2001
Cimzia^TM^	PEG	Interferon α2b/proteic (Chron’s disease)	2008
Omontys^TM^	PEG	Peginesatide acetate/peptidic (Anemia)	2012
Xyotax^TM^	Polyglumex	Paclitaxel/anthracycline (Lung cancer, ovarian cancer)	Phase III
Puricase^TM^	PEG	Uricase/proteic (Hyperuricemia)	Phase III
Mylotarg^TM^	Anti-CD33 monoclonal antibody	Ozogamicin/calicheamicins (Leukemia)	2000
Zevalin^TM^	Anti-CD20 monoclonal antibody	Yttrium-90/radioactive material (Non-Hodgkin’s lymphoma)	2002
Bexxar^TM^	Anti-CD20 monoclonal antibody	Iodine-131/radioactive material (Non-Hodgkin’s lymphoma)	2003
Kadcyla^TM^	Anti-CD37 monoclonal antibody	Emtansine/maytansinoid (Breast cancer)	2013
Opaxio	Paclitaxel covalently linked to solid NPs of polyglutamate	Paclitaxel (Metastatic breast cancer)	2012
Cimzia	Pegylated antibody fragment	Certolizumab pegal (Crohn’s disease, rheumatoid arthritis, psoriatic arthritis, spondylitis)	2008 2009 2013 2013
Plegridy	Pegylated IFN-B1 protein	Interferon B (Multiple sclerosis)	2015
Adynovate (Baxalta)	Pegylated factor VIII	FACTOR VIII (Hemophilia)	2015
Zilretta	Triamcinolone acetonide with a polylactic-co-glycolic acid matrix microsphere	Osteoarthritis of the knee	2017
Rebinyn	Coagulation factor IX GlycoPEGylated	Control and prevention of bleeding in perioperative setting for hemophilia B patients	2017

##### Natural polymers

Natural polymers possess abundance of functional groups, amenable to modifications via chemical or biochemical routes that result in the generation of different types of biopolymer-based materials (Nitta and Numata, 2013; Abedini et al., 2018; Sofi et al., 2018). Among these natural polymers, polysaccharides constitute an important class of polymers which are being used more frequently for various biomedical applications. Polysaccharides are carbohydrate polymers of monosaccharide-based repeating units connected through glycosidic linkages. Their source of production is quite diverse; hence, these have different structures and properties, a wide variety of reactive functionalities, different chemical compositions, and molecular weights (Nitta and Numata, 2013). Based on their functional groups, these have been divided into two main categories, viz., non-ionic (dextrin, pullulan, dextran) and ionic polysaccharides (heparin, chitosan, alginate, etc.). Polysaccharides are considered to be highly stable, safe, non-toxic, biodegradable, hydrophilic, and biocompatible. By tethering lipophilic moieties on the polysaccharides, the resulting conjugates can readily self-assemble into micelles in aqueous solutions and can potentially be used for drug delivery applications. Some of the polysaccharides possess certain bioactive groups which can act as targeting moieties. HA can act as ligand for targeting receptors present on the endothelial cells of liver and certain cancer cells. Self-assembled nanostructures of amphiphilic HAs have been highly investigated as active targeting agents in drug delivery (Cho et al., 2012). Self-assembled structures of modified cellulose, chitosan, and pullulan-based polysaccharides have also been used for colon targeting. These polymers promote drug absorption due to enhanced mucoadhesion in the small intestine. Similarly, amphiphilic heparin-based systems have also shown to reduce tumor size and blood vessel formation in tumor area (Niers et al., 2007). The most commonly and extensively used polysaccharides, namely, alginate, chitosan, and dextran, have been described here along with their therapeutic advantages.

###### Alginate

Alginate (sodium salt) is a water soluble polysaccharide which is made up of 1–4 linked α-L-glucuronic acid and α-D-mannuronic acid in alternating order. Modification of alginate produces diverse polymers which behave in different manners under physiological conditions. As biodegradability of polymer is improved by oxidation of hydroxyl group, sulfonation increases blood compatibility (Kumar et al., 2004). Self-assembled PEG derivatives of alginate have shown significant improvement in hypocalcemia efficacy in rats by enhancing the oral delivery of calcitonin (Li et al., 2012). Recently, phenylalanine ethyl ester modified alginate self-assembled nanoparticles showed good *in vitro* cellular uptake efficiency and biocompatibility profile in human intestinal cell lines (Zhang P. et al., 2019). Ayub et al. (2019) synthesized cysteamine conjugated disulfide crosslinked sodium alginate nanoparticles by layer by layer self-assembly mechanism to get better delivery of an anticancer drug, PTX, for colon cancer. Further, the alginate nanoparticles have been used for antigen delivery also. Antigen-BSA encapsulated polylysine-sodium alginate nanoparticles were formed by process of self-assembly using electrostatic interactions between oppositely charged polyelectrolyte complexes. These particles showed sustained release behavior of vaccine and enhanced cellular uptake without imparting cytotoxicity *in vitro* (Yuan et al., 2018). The self-assembled alginate-based nanoparticles have been used in treatment of multidrug resistant tumors (Kumar et al., 2019) and in combinational chemotherapy (Zhang et al., 2017) as stimuli responsive (redox and light responsive) nanoparticles. Bazban-Shotorbani et al. (2016) synthesized alginate nanogels via microfluidics with tunable pore size and these nanogels were used for protein delivery.

###### Chitosan

Chitosan modifications and their use in delivery applications of various therapeutic molecules have been extensively reviewed in the literature (Coviello et al., 2007; Wasiak et al., 2016; Wang et al., 2017a; Quiñones et al., 2018). Chitosan, an unbranched linear polysaccharide, is made up of β-(1-4)-linked D-glucosamine (deacetylated unit) and *N*-acetyl-D-glucosamine (acetylated unit). It is produced from the skeleton of shellfish, including shrimp, lobster, and crab. It is used in various medicinal formulations such as filler in tablets, controlled-release drugs, and to improve the solubility of drugs. Self-assembly of the modified chitosans into micelles in aqueous solution with hydrophobic pockets has been used to entrap various anti-tumor therapeutics such as PTX, doxorubicin, and camptothecin (Quiñones et al., 2018). Recently, Trummer et al. (2018) synthesized *N*-benzyl-*N*,*O*-succinyl chitosan, *N*-naphthyl-*N*,*O*-succinyl chitosan, and *N*-octyl-*N*,*O*-succinyl chitosan-based self-assembled nanocarriers and successfully co-ordinated to antitumor drug cisplatin and evaluated the efficacy of these nanocarriers *in vitro* in human carcinoma cell line HN22 and HN29. The results showed high efficacy of *N*-benzyl-*N*,*O*-succinyl chitosan-mediated cisplatin delivery. They observed that the encapsulated formulation was less cytotoxic and caused lower cisplatin-induced renal cell death but it exhibited greater apoptosis in HN22 cells as compared to native cisplatin. Besides, the formulation provided long-lasting treatment with reduced nephrotoxicity. Chen et al. (2019) prepared polyelectrolyte complexes via self-assembly of opposite charged alginate-coated nanoparticles and chitosan nanoparticles and used this complex for pH sensitive controlled release of insulin.

###### Dextran

Dextran is a polymer formed from joining of glucose units through α-1,6-linkages with branch points at α-1,2, α-1,3, and α-1,4 linkages. It is non-toxic, highly biocompatible, and could be widely used in medicinal products including development of drug-delivery systems (Wasiak et al., 2016). It has been extensively used as a supplementary material to prevent the formation of blood clots by reducing blood viscosity and iron-dextran conjugates have been applied for fulfilling the iron deficiency. Derivatives of dextran have also been used as the source of biocompatible hydrogels for drug delivery applications to attain regulated and sustained drug release profile for longer time periods (Coviello et al., 2007). Wang et al. synthesized dextran nano-hydrogel by conjugating polyacrylic acid via disulfide crosslinking to dextran. The anticancer drug (doxorubicin) was conjugated to Dex-SS-PAA. The results showed that these nanohydrogels exhibited stimuli (pH and redox) responsive drug release behavior as well as greatly reduced the toxicity of free doxorubicin, and inhibited the growth of MDA-MB-231 tumors (Wang et al., 2017a). In another recent study, folic acid-dextran conjugates were synthesized which showed pH responsive self-assembly behavior. This conjugate self-assembled into nanoparticles at pH 7 and dissociated at pH > 9. The anticancer drug, doxorubicin, was efficiently entrapped in these particles and exhibited targeted drug delivery *in vitro* with enhanced antitumor activity in 4T1 subcutaneous tumor bearing mouse model (Tang et al., 2018). The modified soy-protein and dextran nanogels have been used for the delivery of riboflavin (Jin et al., 2016).

###### Cyclodextrins (CDs)

Cyclodextrins are oligomers of glucose consisting of six, seven, and eight glucose units in α-, β-, and γ-CDs, respectively. The exterior of the cup-shaped molecule is hydrophilic while the internal part is hydrophobic, thus, they are readily soluble in aqueous environment and they can include small, hydrophobic “guest” molecules in their interior and thus forming inclusion complexes (Muankaew and Loftsson, 2018). Due to their inherent biocompatible nature, FDA approved their use in pharmaceutical formulations as solubilizing agents. CD derivatives are synthesized by replacing hydroxyl group on CDs with desired functional groups. The natural biocompatibility and self-assembling attributes of CDs have made them efficient nanocarriers for drug and gene delivery. These molecules can form diverse nanostructures such as nanoparticles, nanospheres, nanogels, nanomicelles, etc. The various modifications have been done on CDs to form amphiphilic derivatives that can self-assemble in aqueous environment and enhance interaction with cell membranes (Simoes et al., 2015). The modified CD amphiphiles can be cationic, anionic, or neutral depending on the groups attached to them. To form sustained drug release carriers, hydrophobic modifications have been done on CDs. He et al. (2013) synthesized acetalated α-CD material that showed pH modulated hydrolysis and pH triggered drug release behavior of encapsulated PTX drug *in vitro*. CDs have also been conjugated to various polymers to improve their physiochemical properties and enhance their drug delivery efficiency (Zhang and Ma, 2013; Zerkoune et al., 2014). Song et al. (2016) prepared β-CD conjugated poly[n-isopropylacrylamide] polymer as a temperature responsive drug carrier. This polymer self-assembled and formed inclusion complexes with PTX drug via host–guest interactions. The enhanced cellular uptake and antitumor effect were observed in cancerous cell lines (Song et al., 2016).

##### Synthetic polymers

Among the commonly used synthetic polymers, block copolymers are a special class of polymers in which two or more blocks of polymers are attached in a regular arrangement. Block copolymers containing two, three, or more blocks are named as diblocks, triblocks, or multiblocks, respectively. PLA, PGA, and their copolymer PLGA, apart from being biodegradable and biocompatible, have been explored for therapeutic delivery and these are approved by the US Food and Drug Administration (Vilar et al., 2012).

Block copolymers are macromolecules which are formed by the linear and/or radial array of two or more dissimilar blocks having different monomer composition to impart amphiphilicity to molecule. The ever-increasing interest in block copolymers has recently arisen due to combinatorial qualities attained by the combination of two different polymers which leads to generation of micellar systems useful for carrying hydrophobic therapeutics.

A variety of amphiphilic copolymers, viz., di-block (A-B) and tri-block (A-B-A) grafted polymers, are being used to form self-assembled nanostructures for different biomedical applications. Among these nanostructures, the micelles are the most common structures formed from these copolymers or block polymers. On dissolving a block copolymer in a solvent, which can be an excellent solvent for dissolving one block and a poor solvent or precipitant for the second block, the copolymer molecules quickly align themselves to attain micellar structure and this phenomenon of micelle formation is reversible also. The most frequently used hydrophilic block is PEG. Other hydrophilic polymers which are commonly used are poly(*N*-vinylpyrrolidone) and poly(*N*-isopropylacrylamide). The core forming hydrophobic blocks which are most frequently used are poly(propyleneoxide), poly ε-caprolactone, polylactic acid (PLA), poly(lactide-co-glycolic acid) (PLGA), poly(L-aspartic acid), poly(L-histidine), poly(β-amino ester), etc. Among these polymers, PLA, PLGA, and PEG are the ones which have been approved or entered the clinical trial phases (Vilar et al., 2012; Felice et al., 2014) (Table 1).

In the last decade, for the fabrication of polymeric nanoparticles, the process of PISA has been used extensively in which polymerization as well as self-assembly occur simultaneously in one vessel to form polymeric nanoparticles. Drug can be encapsulated during the PISA process of nanoparticle formation as well as post-PISA process (Zhang W.J. et al., 2019).

###### Polylactic acid (PLA)

Poly(D,L-lactic acid) is biodegradable polyester used in the fabrication of stents, implants, and various other medical devices (Hoffman, 2008). On hydrolysis, it degrades into monomeric lactic acid, which is also produced during anaerobic respiration in living beings. The polymer, characterized by its inherent viscosity, is dependent on its chain length/molecular weight. A controlled release of the entrapped therapeutic is also dependent on the PLA chain length. PLA is available commercially as Lupron Depot and Risperdal Consta in the form of microparticles. Among PLA matrices, the PLA-PEG micelles have extensively been used in drug delivery applications. For instance, Genexol PL^TM^ is PTX encapsulated PLA-PEG micelles. It is clinically approved in South Korea and Europe (Kim et al., 2004); however, in United States, it is still under phase II clinical trials. Amphotericin B was also encapsulated in PLA micelles and sustained drug release was observed. PLA-based micelles have been used in other drug delivery formulations also (Liu et al., 2008). Apart from these, PLA-based nanoparticles have also been used for entrapment of nucleic acid and their delivery (Munier et al., 2005). Several PLA-based nanostructures are under pre-clinical investigation.

###### Poly(lactic-co-glycolic acid) (PLGA)

Poly(D,L-lactic-co-glycolic acid) is made up of two polymers, i.e. lactic acid and glycolic acid, which on hydrolysis yields biodegradable metabolite monomers, i.e. lactic acid and glycolic acid. These biodegradable metabolites are involved in several biochemical and physiological cycles in the living systems displaying minimal systemic toxicity. Degradation rate of PLGA highly depends on its molecular weight and monomer ratio (Danhier et al., 2012). Till now, PLGA-based therapeutic delivery systems have not been approved but certain PLGA-based systems are under pre-clinical and clinical trials. PLGA-based nanostructures are primarily used in the entrapment of lipophilic antitumor therapeutics, viz., PTX, vincristine sulfate, doxorubicin, curcumin, tetrandrine, etc. (Que et al., 2019). In one of the latest reviews, the industrial and scientific aspects of PLGA nanoparticles have been highlighted (Qi et al., 2019).

###### Polyethylene glycol (PEG)

Polyethylene glycol is a polyether, a non-biodegradable hydrophilic polymer with a variety of applications in pharmaceutical and biomedical areas (Hutanu et al., 2014). PEG helps in increasing the dispersibility of the attached molecules. It has been used in the preparation of polymer-drug conjugates and provides stabilization as well as imparts stealth properties to the so formed DDS.

Polyethylene glycol polymers with reactive functionalities at their termini have been demonstrated to exhibit wide variety of applications. Bi-functional and mono-functional derivatives are also being used as crosslinkers and linkers or spacers. PEG-based carriers for drug delivery such as micelles, nanoparticles, dendrimers, and liposomes are better than PEG conjugates of the drugs (Hutanu et al., 2014).

###### Dendrimers

Dendrimers are the specialized macromolecules which offer regular and highly branched three-dimensional structures. Their unique structures show high density of functionalities at the periphery of the molecules. For instance, dendrimers with peripheral amines allow efficient condensation of negatively charged nucleic acids while the tertiary amines in the core remain available for playing an important role during endo/lysosomal acidification which enables more efficient endosomal release. Dendrimers consist of three major architectural components: a core, inner shell, and an outer shell. These can be synthesized in two ways to have different functionality in each of these components to modulate various properties such as solubility, thermal stability, and attachment of compounds for particular applications (Thota et al., 2015). A dendrimer is typically symmetric around the core. Its structure provides relatively easy access to control their size, composition, and chemical reactivity very precisely. The degree of branching is expressed in the form of generation of the dendrimers. The size and surface charge on dendrimers vary with the number of “generations” during synthesis. Because of the presence of large number of tertiary amines, PAMAM dendrimers act not only as proton sponges in gene delivery applications but also along with carbon skeleton, they have been used as drug carriers simultaneously (Salzano et al., 2016; Abedi-Gaballu et al., 2018; Li et al., 2018). Haensler and Szoka (1993) reported for the first time the use of PAMAM dendrimers in gene delivery. They showed that the sixth-generation dendrimer was almost 10-folds better than lower generation ones. Based on this study, PAMAM dendrimers have recently been used in several *in vivo* and *in vitro* gene delivery applications and found to be biocompatible (Maruyama-Tabata et al., 2000; Yang et al., 2015; Araújo et al., 2018). PAMAM dendrimers have a well-defined size and shape but offer limited flexibility. Therefore, attempts have been made to hydrolytically cleave some of the amide bonds in the inner part. Breaking of some of the branches of dendrimers in the core enhances the flexibility and the resulting molecules are known as activated dendrimers. Although activated and inactivated dendrimers were found to form complexes with DNA by electrostatic interactions and mediated transfer of bound DNA into eukaryotic cells, the overall transfection efficiency of activated dendrimers was found to be two to three times higher than the inactivated (native) dendrimers. The fractured or activated dendrimers not only showed the greater flexibility to interact with plasmid DNA but their solubility also enhanced and presented less tendency to aggregate. This enhanced flexibility of activated dendrimers showed better endosomal release of the DNA and subsequently, the transfection efficiency. SuperFect (from QIAGEN, Hilden, Germany) is an example of commercially available efficient transfection reagents based on the fractured G-5 PAMAM dendrimer. In another attempt, the peripheral amines of PAMAM-G4 were converted into guanidinium (Gn) and tetramethylguanidinium (TMG) moieties. Although these modified dendrimers did not display cytotoxicity in various mammalian cells, higher transfection efficiency was observed only in case of guanidinium-PAMAM-G4 (Yadav et al., 2014b). Somani et al. have investigated the effect of pegylation (2 and 5 kDa) on G-3 and G-4 diaminobutyric polypropylenimine dendrimers. Cytotoxicity decreased significantly in these modified dendrimers without compromising DNA condensability; however, enhanced gene expression was found in G-3 and G-4 daminobutyric polypropylenimine dendrimers conjugated to 2 kDa PEG in cell specific manner (Somani et al., 2018). Further, Gao et al. studied structure activity relationship to design efficient gene delivery vectors. They demonstrated that both hydrophobic modification and density of amines modulate the gene transfer ability of synthetic vectors (Gao et al., 2018).

Subsequently, several hydrophobic modifications have also been incorporated in dendrimers to make them amphiphilic which can self-assemble and be used as delivery vectors (Bolu et al., 2018). Han et al. (2017) developed an amphiphilic conjugate of PAMAM dendrimer by conjugating hydrophobic PLA which on self-assembly formed core-shell nanostructures in which 5-FU and doxorubicin were entrapped efficiently for combinatorial anticancer therapy. This nanomicelle system showed synergistic antitumor effect on MDA-MB 231 tumor cell line and MDA-MB 231 xenograft mice model (Han et al., 2017). In another report, an amphiphilic block micelle was synthesized by conjugating hydrophobic block of linear poly e-caprolactone polymer with hydrophilic part of methoxy terminated PEG decorated G-3 polyester dendron (WooáBae, 2011). They further explored the effect of peripheral functional group such as amine, carboxylic, and acetyl using –NH_2_, –COOH, –COCH_3_ group terminated PEG chains instead of methoxy terminated PEG used in their earlier study (Pearson et al., 2012). The group used this amphiphilic micellar system to encapsulate and deliver anticancer drug, endoxifen. Dendron micelle system containing carboxy terminated PEG showed the highest potential to deliver the drug across skin layers among the tested systems (Yang et al., 2014). This research group further evaluated the effect of density of targeting moiety, folic acid, and PEG length on these dendritic micelles in terms of interaction with cells (Pearson et al., 2016). There are lots of preclinical studies on dendrimers-based drug delivery; however, the clinical ones are very few. A dendrimer drug formulation, DTXSPL8783, for advanced cancer treatment, is currently undergoing clinical phase 1 trial (Caster et al., 2017; Ventola, 2017), while another dendrimer-based antiviral product, Vivagel from Star Pharma, is in phase III trials for bacterial vaginosis (Ventola, 2017). Some of the nanoengineered polymeric systems either approved by FDA or in the advanced clinical phases are listed in Table 1.

#### Self-Assembling Small Molecules

The self-assembled nanostructures formed from the small peptides (Fleming and Ulijn, 2014; Panda and Chauhan, 2014), lipid-based systems (Li et al., 2015), and other small molecules (Xing and Zhao, 2016) can be used as carriers for different therapeutic molecules (Wang et al., 2017b; Liu et al., 2018; Yang et al., 2018). The small molecules can be produced easily as compared to the larger ones and act as efficient and economical alternatives to the large molecule-based systems. Moreover, different small peptides can be combined with diverse synthetic molecules thus producing tailored nanoscale engineered biomaterials that can be used as carriers of genetic materials, drug molecules, and antimicrobial agents. Now-a-days, carrier free and self-assembled small molecule-based nano-DDS are also being developed with the aim to eliminate the issue of undefined metabolism and clinical safety of the carriers (Guo et al., 2017; Jiang et al., 2017; Zheng et al., 2019). Recently, Guo et al. have demonstrated the efficacy of a carrier free system by developing a theranostic nanodrug delivery formulation for NIR imaging and chemotherapy. In this system, indocyanine green, ursolic acid, and PTX formed a self-assembled system via aromatic pi-pi and electrostatic interactions (Guo et al., 2017). Similarly, another carrier free nano-DDS was developed in which anticancer drug doxorubicin and ursolic acid were co-assembled by pi-pi stacking, hydrophobic, and electrostatic interactions, and modified with HER-2 aptamer for targeting to HER-2 receptors overexpressing cancer cells (Jiang et al., 2017).

##### Lipids

This group of carrier materials comprise of cholesterol and phospholipids as the key constituents. Phospholipids, the constituent of all cell membranes as well as the major component of liposomes, are mainly one or two fatty acids linked to glycerol or sphingosin with a phosphate head group which impart amphiphilicity facilitating the formation of bilayered membranes in liposomes. It is reported in the literature that the ordinary amphiphiles have critical micellization concentrations in the range of 10^–2^–10^–4^M, whereas CMC of phospholipids is four to five times smaller, thus these materials have extremely low water solubility. As a consequence of this, they have high stability after administration in comparison to micelles (Felice et al., 2014). Among the natural and anionic phospholipids, the phosphatidylcholine, which is the most studied lipid, present in both plants and animals, consists of a phosphate and quaternary ammonium group. Among the natural anionic phospholipids, phosphatidic acid, phosphatidylglycerol, phosphatidylserine, and phosphatidyl ethanolamine are the most commonly used ones.

The natural cationic lipids such as the stearylamine are mainly used for encapsulation of nucleic acids. The synthetic phospholipids such as dimyristoyl-phosphatidylcholine, dipalmitoylphosphatidylcholine, and DSPC are also used. Natural phospholipids are preferred over synthetic ones as they show greater chemical biostability against phospholipases, esterases, bile salts, and serum proteins thus imparting the greater thermodynamic stability to the vesicles against alkaline pH, high temperature, and oxidative stress conditions. On the other hand, liposomes permeability can be controlled in a better way using synthetic lipids. In biological environment, the phospholipids are degraded by lipolysis and thus result in low toxicity. The fluidity of the liposomal membrane is influenced by the viscosity of the phospholipids which is regulated either by using phospholipids possessing elevated phase shift temperatures, or through insertion of cholesterol molecules. The second most commonly used lipid in nanoengineered DDSs is cholesterol, which is responsible for reducing the permeability of hydrophilic molecules by increasing the stability of liposomal membrane (Felice et al., 2014). Kirby et al. studied the effect of cholesterol on liposomal membranes prepared using natural phospholipids and found that cholesterol containing liposomal membranes were more stable in comparison to cholesterol deficient membranes (Felice et al., 2014) in blood. Deng et al. (2018) and Massiot et al. (2019) developed X-ray radiation triggered (Deng et al., 2018) and photo-triggered (Massiot et al., 2019) liposomal systems for sustained release of the drug molecules (Pattni et al., 2015; Deng et al., 2018; Massiot et al., 2019). A great development has been achieved on liposomal technology advancement; however, their full potential is yet to be explored, as successful bench to bedside applications are very few. So, there is still the need of further development of liposomal technology for advancement in therapeutic delivery systems (Pattni et al., 2015).

Most of the commercialized liposomes have been used to encapsulate anticancer drugs. Among them, Myocet^TM^ and Doxil^TM^, which encapsulate doxorubicin, are the most efficacious formulations (Felice et al., 2014) (Table 2).

**TABLE 2:** List of nanoengineered liposomal therapeutic delivery systems either approved by FDA or in advanced clinical trials (Felice et al., 2014; Bulbake et al., 2017; Patra et al., 2018).

**Product name**	**Carrier material composition**	**Drug/type of drug (Disease)**	**Approval year/phase**
Doxil^TM^	HSPC:cholesterol:PEG 2000-DSPE (56:39:5 molar ratio)	Doxorubicin/anthracycline (Various types of cancers)	1995
DaunoXome^TM^	DSPC and cholesterol (2:1 molar ratio)	Daunorubicin/anthracycline (HIV)	1996
Ambisome^TM^	HSPC:DSPG:cholesterol:(2:0.8:1 molar ratio)	Amphotericin B/polyene (Fungal infections)	1997
Depocyt^TM^	DOPC, DPPG, cholesterol, and triolein	Cytarabin/nucleoside (Lymphomatous meningitis)	1999
Visudyne^TM^	Verteporphin:DMPC and EPG (1:8 molar ratio)	Verteporfin/benzoporphyrin (Macular degeneration)	2000
DepoDur^TM^	DOPC, DPPG, cholesterol, and triolein	Morphine/opiate (Severe pain)	2004
Octocog^TM^	Phospholipids	α Factor VIII/proteica (Hemophilia)	2009
Marqibo^TM^	SM:cholesterol (60:40 molar ratio)	Vincristine/alkaloidsulfate (Hodgkin lymphoma)	2012
Myocet^TM^	EPC:cholesterol (55:45 molar ratio)	Doxorubicin/anthracycline (Metastatic breast cancer)	Phase III/2000
Thermodox^TM^	Low phase transition temperature phospholipids	Doxorubicin/anthracycline (Metastatic malignant melanoma, liver cancer)	Phase III
Onivyde^TM^	DSPC:MPEG-2000:DSPE (3:2:0.015 molar ratio)	Irinotecan (Pancreatic cancer CRC, lung, glioma)	2015 (approved for pancreatic cancer), Phase II, III trials for other cancers
Mepact	DOPS:POPC (3:7 molar ratio)	Mifamurtide (Non-metastaic osteosarcoma)	2004
Abelcet	DMPC:DMPG (7:3 molar ratio)	Amphotericin B (Invasive several fungal infections)	1995
Amphotec	Cholesteryl sulfate	Amphotericin B (Severe fungal infections)	1996
Exparel	DEPC, DPPG, cholesterol, tricaprylin	Pain managment	2011
Epaxal	DOPC:DOPE (75:25 molar ratio)	Hepatitis A	1993
Inflexal	DOPC:DOPE (75:25 molar ratio)	Influenza	1997
Vyxeos (CPX-351)	DSPC:DSPG:cholesterol (7:2:1 molar ratio)	Acute myeloid leukemia	2017

Doxil is the first liposomal formulation of anticancer drug, doxorubicin, permitted by the FDA (United States) in 1995 for AIDS associated with Kaposi’s sarcoma (Northfelt et al., 1998). In this case, the stealth liposomal carrier consists of HSPC, cholesterol, and PEGylated phosphoethanolamine. By encapsulating doxorubicin into stealth liposome carriers, both the circulation half-life of drug and its accumulation in tumor environment were got enhanced. Doxorubicin, an anthracyclin, is currently being used against various carcinomas and exerts its effect by inhibiting DNA and RNA synthesis but it causes side effects such as severe myelo-suppression and cardiotoxicity (Felice et al., 2014). These side effects were reduced to an extent when doxorubicin was entrapped in liposomes (Fan and Zhang, 2013). Initially, doxorubicin, encapsulated in multilamellar liposomes by passive entrapment, was found to be unsuccessful because of fast release of the drug followed by rapid clearance by phagocytic system of the body. Then active drug loading technique was employed to enhance the drug encapsulation content and stability which resulted in two formulations, Myocet^TM^ and Doxil^TM^. In Myocet^TM^, doxorubicin was encapsulated by a pH gradient, while in Doxil^TM^, potential gradient was used to load the drug molecules. Myocet^TM^ comprises cholesterol and EPC while Doxil^TM^ contains cholesterol and hydrogenated soya phosphatidylcholine. Of these two formulations, PEG coating in Doxil^TM^ improved its pharmaco-kinetic profile over Myocet^TM^. Both Myocet^TM^ and Doxil^TM^ showed significant reduction in the toxic effects of doxorubicin (Hofheinz et al., 2005).

Other liposomal drugs clinically approved are Ambisome^TM^, in which Amphotericin, an antifungal drug is loaded, Depodur^TM^, in which morphine, an analgesic, is loaded and Visudyne^TM^, in which verteporfin, a drug for treatment of macular disintegration, is loaded. Besides, there are a number of other liposomal systems which are under phase II and III clinical trials. Liposome-based formulations in clinical trials are more than other types of nanoengineered DDSs. Among these, ThermoDox^TM^ is a temperature-sensitive liposomal formulation encapsulating doxorubicin. ThermoDox^TM^ comprises DPPC, MSPC, and DSPE-MPEG-2000 (Poon and Borys, 2009), the synthetic phospholipids. ThermoDox^TM^ releases its doxorubicin content under the influence of temperature, i.e. above 39.5°C as this formulation has comparatively low Tm. Tm, phase Tm, is the temperature needed to induce change in the arrangement of lipid chains. At this temperature, the aligned gel phase structure changes into the non-aligned liquid crystalline phase structure. In the living system, this temperature (Tm) can be attained by heating the tumor with radio-frequency electromagnetic waves.

##### Small peptides

Liposomes are associated with some technological issues such as stability, reproducibility, poor drug loading, and insufficient control over drug release (Torchilin, 2005). The basic idea of fabricating nanostructures from peptide-based biomaterials came from the literature survey that showed that in certain diseases such as Parkinson’s, Alzheimer’s, and in Prion-related ones, the self-organized tubular shaped proteins were formed from otherwise soluble amphiphilic proteins in the cells (Gazit, 2004). These findings led the way to divert attention and focus researches on investigating self-assembly of peptides to form ordered structures. Peptides possess several inherent properties such as biocompatibility and biodegradability which make them very useful building blocks for forming self-assembled nanostructures for various biomedical applications (Panda and Chauhan, 2014). Furthermore, in case of peptides, the versatility of the chemical design and their capability to assume highly ordered organized structures offers chance of manipulating the final assembly by controlling structural features at the nanometric range. The properties of peptides such as sequence-based unique self-organization and recognition provide them a function of acting as a significant signaling molecule and key building component of living beings. A range of self-organized nanostructures with distinct shapes such as fibrous, tubular, rod, particles, and various other shapes are also formed through self-assembly of peptides (Gazit, 2004; Panda and Chauhan, 2014; Prakash Sharma et al., 2015). In the past few years, research on medium sized peptides, small peptides, ultra small peptides, and peptide-conjugates have opened up new avenues for designing and synthesis of peptide-based self-assembled nanostructures for different biological applications (Fan et al., 2017; Guyon et al., 2018; Ni and Zhuo, 2019; Tesauro et al., 2019). These self-assembled peptide-based nanomaterials can be designed with ease to act as new scaffolds to mimic various biomaterials, tissues, etc. The usefulness of di- or tri-peptide based self-assembled nanostructures has been reported by many research groups. A variety of peptides such as surfactant like peptides, amphiphilic peptides, bolaamphiphiles, peptides containing α-helix, β-sheets, and β-turns, as well as cyclic peptides have been nanotized and studied in detail the process involved in their conversion. Peptides are generally labile to enzymatic degradation which limits their use as therapeutic delivery agents but this limitation has been circumvented, to an extent, by incorporation of non-coded residues in peptide sequences (Gupta et al., 2007).

The self-assembled peptide-based nanostructures, i.e. nanotubes were developed for the first time by Ghadiri et al. using cyclic polypeptides, cyclo-[-(L-Gln-D-Ala-L-Glu-D-Ala)_2_-], containing even number of D- and L-amino acids alternatively (Hartgerink et al., 1996). These cyclic peptides self-assembled to form nanotubes via intermolecular hydrogen bonding in which side chains of the amino acids lied toward the exterior surface and formed a hollow tube type arrangement. The cyclic peptide-based nanotubes have been used as antimicrobial agents, biosensors, catalysts, etc. (Brea et al., 2010).

A large number of peptides self-organize via formation of β-sheeted secondary structures. β-Sheeted peptides self-assemble to form diverse supramolecular architectures such as nanoribbons, nanotubes, monolayers with nanoscale order, etc. (Reches and Gazit, 2003; Ashkenasy et al., 2006). The 16-amino acid residues containing peptides, RADA-16 I and RADA-16 II, formed β-sheeted structures that produced nanofibrous network followed by pH-responsive hydrogels (Holmes et al., 2000) via self-assembly. The stimuli responsive hydrogel network formation by these peptides can also be enhanced by increasing ionic strength or altering the pH of the assembling environment. These peptides are now available commercially with the name, PuraMatrix (3DM, Inc., Cambridge, MA, United States).

The surfactant-like peptides also self-assemble to form nanostructures (Vauthey et al., 2002). These seven to eight amino acids long surfactant-like peptides (A_6_D_1_, V_6_D_1_, V_6_D_2_, L_6_D_2_) have similar properties as observed in biological surfactant molecules. They also contain a negatively charged hydrophilic head group at C-terminus, i.e. aspartic acid, and the hydrophobic amino acids, i.e. alanine, valine, or leucine formed the part of lipophilic tail. The N-terminus of the peptides was acetylated to create a neutral moiety, which facilitated self-assembly via electrostatic and hydrophobic forces (Tsonchev et al., 2008).

Amphiphilic peptides are formed via hydrophilic peptide forming head group and hydrophobic alkyl tail. The hydrophobic alkyl end helps in arrangement of the hydrophilic part to self-assemble in different higher order structures. These amphiphilic peptides self organize to form diverse morphological structures with nanodimensions such as micelles, vesicles, or tubules (Panda and Chauhan, 2014).

Bolaamphiphiles (KA_6_K, KA_4_K, KA_8_K) are amphiphilic molecules made up of two hydrophilic groups flanked by hydrocarbon chains. In these peptides, β-sheet H-bonding interactions result in formation of a variety of structures such as nanofibers, nanorods, nanotubes, nanoribbons, nanospheres, etc. (Panda and Chauhan, 2014). However, in case of nanostructure formation by these large linear peptides, cyclic and dendritic structures, the related expense and complexity of synthesis restrict the use of these peptides practically. In addition to this, these peptides are also not stable under enzymatic exposure which hampers the use of these peptides in biological applications.

Very short peptides such as di-, tri-, tetra-, and pentapeptides also self-assemble to form diverse nanostructures (Panda and Chauhan, 2014). These short peptide fragments were carefully studied in order to find out the minimum sequence required for amyloid formation. In amyloid fibrils polypeptide, a hexapeptide fragment NFGAIL (hIAPP22–27) of the islet amyloid polypeptide (IAPP) formed the well-organized amyloid fibrils which were alike to amyloid fibrils of complete polypeptide. Further, it was found that a pentapeptide fragment FGAIL (hIAPP23–27) of the IAPP also formed a fibrillar structure. Similarly, AILSS fragment was also discovered as strong amyloidogenic region of IAPP. Another peptide part, KLVFF of the amyloid β-peptide Ab-42, self-organized in saline buffer forming hydrogel. A pentapeptide sequence in human calcitonin, i.e. DFNKF, also formed the well-ordered amyloid fibrils similar to those observed in case of full length polypeptide. All these observations revealed that peptides which form amyloid fibers have a shorter sequence of amino acids which can also self-organize to form amyloid fibrils similar to those formed by complete peptide. Furthermore, these research studies recommended the significant role of aromatic amino acids in formation of amyloid fibrils. Further research on amyloid like fibrils suggested that α-amino isobutyric acid (U) containing small peptide, i.e. Boc-AUV-OMe, Boc-AUI-OMe, and Boc-AGV-OMe form β-sheet structures which self-organize in amyloid like fibrils (Panda and Chauhan, 2014).

Dipeptides also self-assemble to form nanostructures was demonstrated for the first time by Gazit et al. using dipeptides, FF (Reches and Gazit, 2003). This dipeptide self-assembled into different nanostructures, i.e. nanotubes/microtubes, nanowires, and nanoforests (Reches and Gazit, 2003; Marchesan et al., 2015). The nanotubes formed by FF dipeptide were thermally stable. They further demonstrated that an incorporation of –SH group in FF resulted in transition from tubular to spherical structures (Reches and Gazit, 2004). There are reports in literature that hydrophobic dipeptides, LL, LF, FL, and IL self-assemble to nanotubes via head to tail (NH_3_^±^OOC) H-bond formation (Panda and Chauhan, 2014). Further reports exist in the literature, which demonstrate that VA, LS, and FF can also form nanoporous structures. The amine and carboxyl terminal modified dipeptides also form self-assembled nanostructures. Fmoc-FF dipeptide form hydrogels with a nanofibrillar structure in aqueous conditions and physical attributes of these hydrogels were found to be better than the hydrogels formed by longer polypeptides. The same research group further evaluated the effect of modification of –NH_2_ and –COOH terminals of FF dipeptides on self-assembly behavior and found that N and C terminal modified dipeptides also form self organized structures in nano-range. Furthermore, the hydrogels were formed from Fmoc protected dipeptides using a combination of glycine, alanine, leucine, and phenylalanine. The physical and structural features of these hydrogels were different, depending on the characteristic of amino acids used in dipeptide sequences. There are numerous reports in literature which suggest that aromatic moieties such as Fmoc and pyrene protected peptides form nanofibrillar hydrogel network due to π-π stacking and hydrophobic interactions. Unsaturated amino acids such as dehydrophenylalanine have also been used to form self-assembled nanostructures (Panda and Chauhan, 2014). The introduction of dehydrophenylalanine provides conformational constrain and proteolytic stability to peptides. The extensive studies on dehydrophenylalanine (ΔF) containing dipeptides have been done by Chauhan et al. where the ΔF was used as C-terminal amino acid and N-terminal amino acid residue was varied with any one of the natural amino acids (Gupta et al., 2007). They found that dipeptides with aromatic amino acid at N-terminus formed nanotubes while the dipeptides having charged amino acid at N-terminus formed vesicles. They also revealed that these dipeptides having hydrophobic groups at their N-terminus give rise to self-assembled structures which can be seen from human eye while the structures formed with hydrophilic N-termini were invisible to naked human eye (Panda and Chauhan, 2014). The dipeptide, F-ΔF, assembled into hydrogels at pH 7.0. The hydrogel formed from F-ΔF dipeptides efficiently entrapped and released drugs, antibiotics, and vitamins. The kinetics of drug release was affected by change in pH and ion concentration (external stimuli). Thus, this system was used as a controlled self-regulated drug release system (Panda et al., 2008). Among the tested dipeptides having charged amino acids at N-termini, EΔF, KΔF, RΔF, and DΔF, the dipeptide, RΔF formed vesicular structures and was easily functionalized with folic acid to target folic acid receptors. These nanostructures showed enhanced cellular uptake in various cancer cell lines, like MDA-MB-231 and HeLa. These folic acid functionalized RΔF nanostructures also encapsulated doxorubicin efficiently. These doxorubicin-loaded nanostructures showed enhanced cytotoxicity toward cancer cells. These nanostructures further showed enhanced targeting and accumulation in tumor tissue in Ehrlich ascitic tumor bearing mice.

**FIGURE 4:**
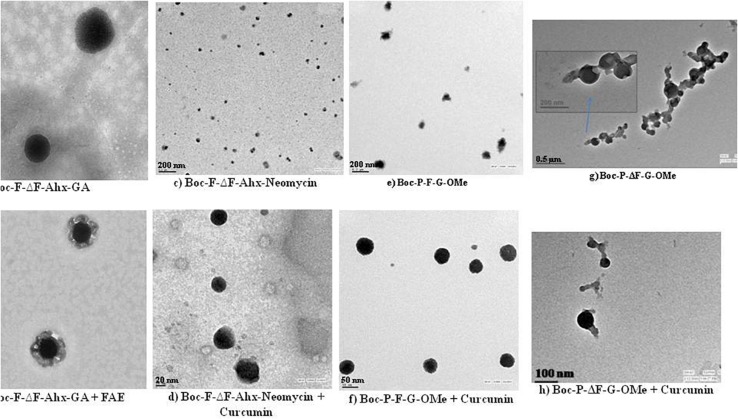
TEM images of **(a)** Boc-F-ΔF-Ahx-GA dipeptide, **(b)** FAE-loaded Boc-F-ΔF-Ahx-GA dipeptide, **(c)** Boc-F-ΔF-Ahx-Neomycin dipeptide, **(d)** Curcumin-loaded Boc-F-ΔF-Ahx-Neomycin dipeptide, **(e)** Boc-P-F-G-OMe tripeptide and **(f)** Curcumin-loaded Boc-P-F-G-OMe tripeptide; **(g)** Boc-P-ΔF-G-OMe tripeptide and **(h)** Curcumin-loaded Boc-P-ΔF-G-OMe tripeptide. (**a** and **b:** Reproduced from Reference (Mahato et al., 2012) with permission from the Royal Society of Chemistry; **c** and **d:** Reproduced from reference (Yadav et al., 2014a) with permission from the Royal Society of Chemistry; **e** and **f:** Reproduced from reference (Yadav et al., 2015) with permission from Bentham Science Publishers; **g** and **h:** Reproduced from reference (Deka et al., 2017) with permission from Elsevier.

In yet another study, Mahato et al. (2012) prepared self-assembled nanostructures in aqueous environment from small glycolated dehydropeptides, Boc-F-ΔF-εAhx-GA and H-F-ΔF-εAhx-GA, wherein glucosamine was attached to peptides via 6-aminohexanoic acid linker. These peptides efficiently entrapped the hydrophobic dyes, eosin and *N*-fluoresceinyl-2-aminoethanol (FAE), in their core (TEM images in Figures 4a,b). At higher concentration, Boc-F-ΔF-εAhx-GA formed hydrogel also. Likewise, Yadav et al. synthesized Boc-F-ΔF-εAhx-Neomycin by conjugating Boc-F-ΔF-εAhx-OH with an aminoglycoside, neomycin, which on self-assembly in aqueous environment formed nanostructures having hydrophobic core and cationic hydrophilic shell. These cationic nanostructures efficiently interacted with pDNA and showed enhanced transfection efficiency in mammalian cells *in vitro*. Besides, these nanostructures efficiently entrapped hydrophobic molecules, eosin and curcumin, in the core of nanostructures which were characterized by electron microscopic imaging (TEM images, Figures 4c,d) (Yadav et al., 2014a). Later on, the same research group fabricated a tripeptide, Boc-P-F-G-OMe, which on self-assembly yielded nanostructures and acted as drug carrier by efficiently encapsulating hydrophobic drug molecules such as eosin, aspirin, and curcumin (Figures 4e,f) (Yadav et al., 2015). This group further synthesized a dehydropeptide, Boc-P-ΔF-G-OMe containing dehydrophenylalanine, an unnatural amino acid, to check the effect of dehydrophenylalanine on the formation of self-assembled nanostructures. The incorporation of dehydrophe instead of Phe improved the encapsulation efficiency of hydrophobic drug, curcumin, in these nanostructures (Figures 4g,h). These nanostructures were further stabilized with vitE-TPGS. These nanostructures showed that incorporation of constrained dehydro amino acid in peptides resulted in the construction of stable nanostructures for the development of nanomaterials with controlled drug release (Deka et al., 2017).

Stimuli-responsive peptide nanostructures have, recently, attracted the attention of the researchers as controlled drug delivery vehicles since these are capable of releasing the drug at the intended sites (Panda et al., 2008). The therapeutic molecule-encapsulated peptide nanostructures can be made to release the therapeutics on exposure to stimuli such as change in pH, temperature, and others. There is a report in the literature where anti-inflammatory prodrug, olsalazine, has been conjugated with a tripeptide derivative and this conjugate self-assembled in water to form supramolecular hydrogels (Li et al., 2010). Moreover, the release of 5-aminosalicylic acid from these hydrogels was made to occur in an organized way, attained by reducing the azo bond which resulted in disassembly of the hydrogels. Moreover, the nanovesicles developed from dipeptides using glutamic acid at C-terminus showed constant behavior over a range of pH. However, these vesicles were responsive to Ca^2+^ ions. In these nanovesicles, anticancer drugs, fluorescent dyes, and various biologically active molecules were entrapped and released in response to calcium ions (Naskar et al., 2011). The peptide nanostructures formed from peptide, Acp-YE (Acp, 3-amino caproic acid), showed stimuli responsive behavior to Ca^2+^ ions concentration and change in pH. The release of encapsulated anticancer drug (doxorubicin) from these vesicular structures was achieved on exposure to Ca^2+^ ions concentration.

Enormous literature exists on self-assembled peptide nanostructures useful for drug delivery applications; however, most of them are under *in vitro* studies. Only few *in vivo* studies have been undertaken and some of them have been listed in Table 3 (Leite et al., 2015; Habibi et al., 2016; Lee et al., 2019).

**TABLE 3:** List of peptide-based self-assembled nanostructures for drug delivery applications.

**Peptide nanostructure**	**Carrier material**	**Drug (Disease)**	**References**
Nanofibers	RAD16-II AcN-(RARADADA)2-CNH2	IGF-1 (Myocardial infarction)	Davis et al., 2006
Nanofibers coated with chitosan	Leucine-enkephalin NH_2_-Y(-O-palmitoyl)GGFL-OH	Enkephalin (Pain)	Lalatsa et al., 2015
Nanofibers coated with chitosan	NH_2_-Y(-O-palmitoyl) GGFLR-OH	Dalargin (Pain)	Mazza et al., 2013
Nanofibers	Palmitoyl-A4G3E3	Camptothecin (Cancer)	Soukasene et al., 2011
Nanofibers	Palmitoyl-V2A2E2K	Dexamethasone (Inflammation)	Webber et al., 2012
Nanofibers	Palmitoyl-G3A4IKVAV	Spinal cord injury	Tysseling-Mattiace et al., 2008
Nanofibers	Palmitoyl-(K)-V3E3SGGGYPVH PST-NH_2_	Bone morphogenetic protein-2 (Spinal arthrodesis)	Lee et al., 2015
Nanoparticles	Polylactide-V6K2(VVVVVVKK)	Doxorubicin, Paclitaxel (Cancer)	Jabbari et al., 2013
Nanotubes	Lanreotide NH_2_-(D)Naph-CY(D)WKVCT-CONH_2_	Acromegaly	Freda et al., 2005; Theodoropoulou and Stalla, 2013

##### DNA nanotechnology-based drug delivery

DNA nanotechnology involves assembly of synthetic DNA fragments into self-assembled nanostructures of different sizes, shapes, and morphology. The basic principle behind DNA nanotechnology is the complementary base pairing. Using this principle, a large number of simple DNA nanostructures have been produced (Hu et al., 2018; Madhanagopal et al., 2018; Kim et al., 2019). Initially, the research on DNA nanotechnology was initiated with the formation of simple topological morphologies which later on advanced to production of DNA tiles, periodic lattices, and 2D and 3D structures. Using this superamolecular DNA technology, wherein interactions such as van der Waals, pi-pi stacking, H-bonding, metal-coordination etc., are involved in DNA self-assembly, different molecules have been encapsulated (Sharma et al., 2018; Ariga et al., 2019). In the last decade, the advent of DNA origami in 2006 by Rothemund’s group expanded the research on DNA nanotechnology from 2D to 3D confirmation forming complex 3D nanostructures of diverse shape and design. In DNA origami, a large natural single-stranded DNA is folded with the help of several chemically synthesized short DNA strands called staple strands to form well-defined 2D and 3D nanostructures. The different types of DNA nanostructures can be fabricated by varying the number and length of staple strands as well as by changing the relative sequence of nucleotides in individual strand. These small staple strands induce the folding of larger DNA strand by annealing with it. The complementary base pairing interaction of DNA scaffold with staple strands help in self-assembling in well defined nanoarchitectures (Hu et al., 2018; Madhanagopal et al., 2018; Sharma et al., 2018).

The self-assembled DNA nanostructures have been used in various biomedical applications such as drug delivery, gene delivery, biosensing, etc. (Ariga et al., 2019). The design and the strategy used to construct these DNA nanoarchitechtonics depend on the type of application these nanostructures are required. The DNA nanostructures, formed via self-assembly approach, are mostly based on sticky end cohesion of DNA strands. The sticky ends are unpaired nucleotide overhangs at the end of DNA molecules. These overhangs are mostly palindromic sequences. The sticky ends are used to combine DNA nanostructures via hybridization of their complementary single strands. Initially, the polyhedral DNA nanostructures were formed by this approach. Subsequently, periodic lattices were formed by tile-based self-assembly approach. With the dawn of DNA origami approach, a lots of 2D and 3D objects were created. DNA origami approach is successfully used to create large nanostructures compared to tile-based approach, as in DNA origami, thousands of nucleotide long scaffold DNA strand is employed. Another strategy has also been used for DNA nanostructures in which single-stranded DNA tiles containing four domains are used to create DNA nanostructures and adjacent tiles bind with complementary parts forming DNA lattices composed of parallel DNA helix (Madhanagopal et al., 2018).

Construction of customized DNA nanostructures is driven by the type of therapy and therapeutic molecules to be delivered. Various types of therapeutic molecules can be delivered, e.g. drug molecules, fluorescent dyes, protein molecules, siRNA, miRNA, CpG sequences, mAB, etc. Fluorescent dyes, viz., fluorescein, cyanine, and rhodamine, have been tagged with self-assembled DNA nanostructures and delivered to cells for different cellular analysis (Hu et al., 2018; Lacroix et al., 2019). Ding et al. have used DNA origami to synthesize 2D DNA triangle and 3D DNA tubes to load anticancer drug, doxorubicin. These DNA origami nanostructures showed enhanced cellular uptake in adenocarcinoma cell lines, MCF-7 and Dox-resistant MCF-7 cells (Jiang et al., 2012; Zhao et al., 2012; Zhang et al., 2014; Mei et al., 2015; Li et al., 2017).

DNA nanostructures have also been used to deliver oligonucleotides, i.e. CpG dinucleotides as vaccine adjuvants for immunotherapy of infectious diseases (Zhang et al., 2015; Wang et al., 2016; Qu et al., 2017; Hu et al., 2018). The CpG motifs are present in bacterial genome and they are recognized as foreign molecules by vertebrate immune system. So these DNA motifs have been used to trigger host immune response as these are recognized by TLR-9 located on endosomes of host membrane of immune system which activates the innate immune pathway of host immune system. Similarly, siRNA and miRNA delivery have also been carried out using DNA nanostructures for gene silencing applications (Lee et al., 2012; Fakhoury et al., 2013; Bujold et al., 2016; Roh et al., 2016; Qian et al., 2017; Nahar et al., 2018). Various types of DNA nanostructures, that have been used as delivery vehicles, are listed in Table 4 (Hu et al., 2018; Madhanagopal et al., 2018). Some of the recent reviews on DNA nanotechnology have described in detail the applications of DNA nanotechnology in drug delivery. Despite enormous advantages of DNA-based nanostructures, their stability *in vivo* is an issue as they are sensitive to cellular environment as well as salt concentration. Moreover, the high cost involved in the synthesis of DNA hampers their large-scale applications in biomedical field (Linko et al., 2015). In an attempt to address this concern, Praetorius et al. (2017) have presented a method to knock down the price of DNA nanostructure synthesis using biotechnological mass production. Although this method is not currently available in every lab, it is expected, in near future, that this cost-effective protocol would overcome this obstacle expanding the scope of DNA nanotechnology in other branches of science and technology.

**TABLE 4:** Self-assembled DNA nanostructures as drug delivery vectors.

**Type of DNA nanostructure**	**Drug (Disease)**	***In vitro***	**References**
Tetrahedron	Doxorubicin (Cancer)	MDA-MB 468, MCF-7 cells	Setyawati et al., 2016
Icosahedron 30	Doxorubicin (Cancer)	Epithelial cancer cells	Chang et al., 2011
Pyramidal nanostructure	Doxorubicin (Cancer)	MDA-MB-231, HepG2, LoVo LoVo-R	Kumar et al., 2016
Triangular prisms	Doxorubicin (Cancer)	MCF-7 breast cancer cells	Chan et al., 2016
Crosslinked junctions	Doxorubicin (Cancer)	CCRF-CEM, Ramos, K562, K562/D	Wu et al., 2013
Concatamers	Doxorubicin (Cancer)	Ramos, CEM cells, mouse model	Zhu et al., 2013b
Nanoflowers	Doxorubicin (Cancer)	Ramos, CEM cells, mouse model, MCF-7, HeLa	Zhu et al., 2013a; Hu et al., 2014
Cocoon	Doxorubicin (Cancer)	MCF-7 cells	Sun et al., 2014
Triangular origami, Rectangular origami, Origami nanotubes	Doxorubicin (Cancer)	MCF-7, MDA-MB-231 cells	Jiang et al., 2012; Zhang et al., 2014
Origami nanorods	Daunorubicin (Cancer)	HL-60 cells	Halley et al., 2016
Origami nanoparticle superstructures	Doxorubicin (Cancer)	U87 cells	Yan et al., 2015

## Conclusion and Future Perspectives

In the last 20 years, tremendous developments have been made in the area of self-assembly of bioactive molecules. Post self-assembly, the nanostructure-based materials are potentially useful and have offered newer tools to revolutionize the area of biological and biomedical sciences. Nanotechnology has significantly contributed toward the realization of targeted and controlled delivery of therapeutics. For their delivery, different types of materials/systems have been developed. Barring a few, many of these materials have their own merits and demerits. Certain materials have been claimed to exhibit biocompatibility but the others that have been developed and are being used showing toxicity and hence proved inappropriate for *in vivo* applications. For example, cationic lipid-based nanostructures are found to activate the immune system. Besides, these are also associated with some technological issues such as stability, reproducibility, low drug loading, encapsulation, and uncontrolled drug leaching problems. Polymeric systems were then developed and evaluated but they were also associated with the similar types of limitations and hence surface functionalization was thought of to improve drug or gene- targeting, which is usually complicated. Similarly, natural polymers elicited unwanted immune reactions and also showed a batch to batch inconsistency, thus *in vivo* performance of these polymers became complex and questionable. Peptides and small molecule-based nanostructures can be good alternatives as carriers for therapeutic delivery as they possess certain characteristics such as good biocompatibility, ease of synthesis, and functionalization. Their self-assembled nanostructures present numerous prospective applications in biomedical field. Beside this, easy stimuli-responsiveness (internal/external stimuli) of self-assembled small molecules makes their role vital in the advancement of therapeutics delivery systems, where the therapeutic release behavior can be better controlled according to the requirements. Thus mild and rapid synthesis conditions, easy dispersibility in aqueous medium, simple functionalization, low production cost, and non-requirement of specialized equipments are some of the advantages which have advocated their promising potential to be used as future candidates for applications such as in drug/gene delivery, diagnosis, imaging, sensors, tissue engineering, bioelectronics, production of biomaterials, healthcare-related systems, etc. Various types of structures can be generated simply by varying the conditions. Thus, this area has emerged as a newer area of research which has shown promising potential. However, there exist several challenges which still need to be addressed in order to make them materials of choice for researchers. Although self-assembly results in the generation of various types of structures such as nanotubes, nanoparticles, nanospheres, nanotapes, nanofibers, nanogels, nanorods, etc., controlling the size of these structures during processing, their behavior under aqueous environment, degree of loading/entrapment of therapeutics and stability, as well as upscaling are still the gray areas where sincere attention of the researchers is required. Besides, studies to establish the biocompatibility and immunogenicity of these nanostructures are lacking.

Self-assembled DNA-origami nanostructure-based drug delivery offers a newer area which has shown tremendous potential in cancer treatment. These structures have been shown to possess stability in cell lysates upto 12 h, while, on prolonged exposures, degradation begins to occur. To improve their stability, several modifications have been suggested. Likewise, optimization in size and shape of these nanostructures reveals their effectiveness during drug release. Because of their multifunctional nature, easy amenability to modifications, biodegradability, as well as biocompatibility, these systems can be developed as safe and efficient drug delivery vectors. However, translation from bench to bedside applications, some crucial aspects are still required to examine in detail such as stability of these nanostructures under different conditions, their efficacy in different types of diseases, comparison of their performance with the commercially available formulations, systemic clearance, morphological parameters during their interactions with the different types of cells, effect of surface charge on their stability during circulation, etc. These investigations are required to ascertain that these systems will provide fascinating and promising solutions to improve the area of human healthcare.

## Author Contributions

SY wrote the manuscript with guidance from AS and PK. PK provided the critical feedback and helped in shaping the manuscript in current form. AS and PK supervised the manuscript.

## Conflict of Interest

The authors declare that the research was conducted in the absence of any commercial or financial relationships that could be construed as a potential conflict of interest.

## Abbreviations

Boc: t-butyloxy carbonyl
CDs: cyclodextrins
CMC: critical micellar concentration
DDSs: drug delivery systems
DEPC: dierucoylphosphatidylcholine
DMPC: dimyristoyl phosphatidylcholine
DMPG: dimyristoyl phosphatidylglycerol
DNA: deoxyribonucleic acid
DOPC: dioleoylphosphatidylcholine
DOPE: dioleoly-sn-glycero-phophoethanolamine
DOPS: dioleoylphosphatidylserine
DPPC: (1,2-dipalmitoyl-sn-glycero-3-phosphocholine)
DPPG: dipalmitoylphosphatidylglycerol
DSPC: distearoylphosphatidylcholine
DSPE: distearoyl-sn-glycero-phosphoethanolamine
DSPE-MPEG-2000: (1,2-distearoyl-sn-glycero-3-phosphoethanolamine-*N*-methoxypolyethyleneglycol-2000)
DSPG: distearoylphosphatidylglycerol
EPC: egg phosphatidylcholine
Fmoc: 9-fluorenylmethyloxy carbonyl
HA: hyaluronic acid
HSPC: hydrogenated soy phosphatidylcholine
MPEG: methoxy polyethylene glycol
MSPC: (1-stearoyl-2-hydroxy-sn-glycero-3-phosphocholine)
PEG: polyethylene glycol
PGA: poly(glycolic acid)
PISA: polymerization-induced self-assembly
POPC: palmitoyloleoylphosphatidylcholine
PLA: poly(D,L-lactic acid)
PLGA: poly(D,L-lactic-co-glycolic acid)
PTX: paclitaxel
RNA: ribonucleic acid
SM: sphingomyelin
Tm: transition temperature.
